# Roles of AMPA receptors in social behaviors

**DOI:** 10.3389/fnsyn.2024.1405510

**Published:** 2024-07-11

**Authors:** Qi Wei Xu, Amanda Larosa, Tak Pan Wong

**Affiliations:** ^1^Douglas Hospital Research Centre, Montreal, QC, Canada; ^2^Department of Psychiatry, McGill University, Montreal, QC, Canada

**Keywords:** aggression, autism spectrum disorder, depression, schizophrenia, social memory, subunit composition, synaptic plasticity

## Abstract

As a crucial player in excitatory synaptic transmission, AMPA receptors (AMPARs) contribute to the formation, regulation, and expression of social behaviors. AMPAR modifications have been associated with naturalistic social behaviors, such as aggression, sociability, and social memory, but are also noted in brain diseases featuring impaired social behavior. Understanding the role of AMPARs in social behaviors is timely to reveal therapeutic targets for treating social impairment in disorders, such as autism spectrum disorder and schizophrenia. In this review, we will discuss the contribution of the molecular composition, function, and plasticity of AMPARs to social behaviors. The impact of targeting AMPARs in treating brain disorders will also be discussed.

## 1 Introduction

Social behaviors refer to interactions between individuals of the same species. Interactions between conspecifics are common in the animal kingdom. Social behaviors also have significant impacts on survival and reproduction. Reproductive success can be facilitated by interactions between mates and parental/maternal behaviors. Affiliative social behaviors, such as cooperation and altruism, are crucial for sharing limited resources within a social group. Finally, agonistic behaviors are important for fighting off predators, protecting offspring, and developing social ranks and hierarchies. Expression of social behaviors is mediated by neural networks that are responsible for the detection, computation, and storage of social information. Not surprisingly, impaired social behaviors have been found in almost all brain disorders. Nonetheless, social behaviors are complex and inherently difficult to examine. More studies are warranted to obtain a better understanding of the biological underpinnings of social behaviors.

Rodents are social animals. They have been commonly used for studying sexual (Ventura-Aquino and Paredes, 2017; Heijkoop et al., 2018), affiliative (Panksepp et al., 2007), and agonistic social behaviors (Scott, 1966). The propensity of interacting with a novel conspecific has been extensively used for studying biological mechanisms of social cognition, such as the neural network for social recognition memory (Tanimizu et al., 2017). These studies have revealed brain regions that are crucial for social behaviors. Since neuronal activity is regulated by the balance of excitatory and inhibitory synaptic transmission, emerging findings have revealed significant roles for glutamatergic transmission, the most common type of excitatory synaptic transmission, in social behaviors.

The α-amino-3-hydroxy-5-methyl-4-isoxazolepropionic acid receptor (AMPAR) is the most common ionotropic glutamate receptor. They mediate fast excitatory synaptic transmission. Due to its ubiquitous expression in the brain, it is not surprising that manipulating AMPAR function results in changes to social behaviors. Injecting AMPAR antagonist systemically (Dannenhoffer et al., 2018), or directly into the hippocampus (Ueda et al., 2021a), prefrontal cortex [PFC, (Marcondes et al., 2020)], and striatum (van Kerkhof et al., 2013), which are brain regions that are crucial for social cognition, resulted in significant alterations in social behaviors. Interestingly, blocking AMPARs in the prelimbic PFC either immediately or 3 hours after social recognition training, impaired social memory formation (Marcondes et al., 2020). However, social memory deficit was induced by inhibiting N-methyl-D-aspartate receptors (NMDAR) immediately after training but not 3 hours later, supporting important roles of AMPARs in both the induction and consolidation of social memory. The contribution of AMPARs to social behaviors is far more complex than the machinery of excitatory transmission. AMPARs are multimeric complexes of subunits that comprise the receptor channel and auxiliary subunits, which mediate its gating and trafficking properties. Findings from genetic modifications of these subunits have revealed subunit-specific roles in social behaviors. In addition, changes in AMPAR expression and function are fundamental for synaptic plasticity, a mechanism pertaining to the experience-dependent changes in social behaviors and the storage of social information. Finally, changes in AMPARs have been commonly associated with animal models (Park et al., 2018; Wu et al., 2020; Ueda et al., 2021b; Abhishek et al., 2022), and human diseases (Salpietro et al., 2019) that exhibit social deficits. Understanding the role of AMPARs in social impairment could reveal novel therapeutic targets for treating deficits in social functioning that are currently lacking effective treatment.

In this review, we will discuss findings of the contribution of different AMPAR subunits to social behaviors. Before we review these findings, we will briefly discuss some basic properties of AMPARs below.

## 2 Molecular composition, function, plastic properties and expression patterns of AMPARs

**FIGURE 1:**
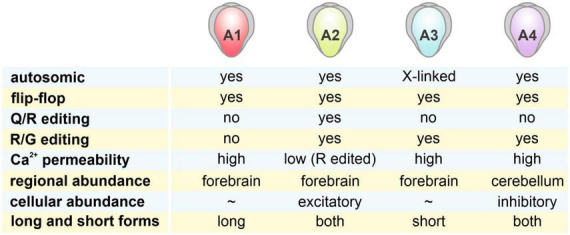
Characteristics of 4 AMPAR subunits. AMPARs are tetrameric complexes of 4 different GluA1-4 subunits, with distinct characteristics shown in the figure. Regional abundance of these subunits was estimated by data from Schwenk et al. (2014). While GluA1-3 are highly abundant in the forebrain regions (cortex + hippocampus + striatum), GluA4 is most highly expressed in the cerebellum. Cellular abundance of these subunits in excitatory and inhibitory neurons was examined in Geiger et al. (1995).

AMPARs are tetrameric complexes from a combination of 4 subunits: GluA1, GluA2, GluA3, and GluA4 (Hollmann et al., 1989; Boulter et al., 1990; Keinanen et al., 1990; Cull-Candy et al., 2006; see also Figure 1). In both rodents and humans, GluA3 and the other GluA subunits can be found on the X chromosomes and autosomes, respectively. While GluA1-3 are widely expressed in the brain, GluA4 expression subsides during development (Zhu et al., 2000). Different subunits are variably expressed in rat brain regions. Over 90% of AMPARs reside in the cortex, the hippocampus, and the cerebellum (Schwenk et al., 2014). Within the hippocampus, over 80% of AMPAR subunits are GluA1- and GluA2-containing while in the cortex and striatum, GluA2 makes up 50% of the population and GluA1 and GluA3 are of similar proportions. Surprisingly, GluA4, which subsides during development in most brain regions, takes up about 64% of AMPAR population in the adult rat cerebellum.

The most preferred way of AMPAR assembly is the combination of two identical dimers (Mansour et al., 2001; Brorson et al., 2004). Earlier findings from an immunoprecipitation study suggested a similar proportion of GluA1/2- and GluA2/3-containing AMPARs in the CA1 region (Wenthold et al., 1996). However, a more recent study using single cell genetic approaches found that over 80% of AMPARs in the CA1 are made up of GluA1/2 (Lu et al., 2009). Nevertheless, these findings suggest the assembly of AMPARs is a regulated, rather than a stochastic, process. AMPAR channels are also bound with auxiliary subunits, such as transmembrane AMPAR regulatory proteins (TARPs) and cornichons (Ziff, 2007; Kamalova and Nakagawa, 2021). These auxiliary subunits are known to regulate the gating and trafficking of AMPARs.

In addition to excitatory principal neurons, inhibitory interneurons comprise 10–15% of the total neuron population in the hippocampus. These interneurons possess a different AMPAR profile from the principal neurons (Geiger et al., 1995). Unlike principal neurons, which express high levels of GluA2, hippocampal interneurons have a higher level of GluA4 expression. The presence of GluA4 and the lower GluA2 expression results in fast gating properties and high Ca^2+^ permeability of AMPARs in interneurons compared to principal neurons.

The functional properties of AMPARs are tightly related to their subunit composition. Compared to GluA3-lacking AMPARs, GluA1-lacking AMPARs have lower conductance (Renner et al., 2017). GluA2 has multiple roles in regulating AMPAR functions. Due to the editing of GluA2 mRNA from glutamine at residue 607 (Q) to an arginine (R), GluA2-containing AMPARs are impermeable to divalent ions like calcium. In addition, unlike the inward rectification properties of GluA2-lacking AMPARs, the presence of GluA2 results in a linear current/voltage relationship and insensitivity to polyamine blockade (Cull-Candy et al., 2006; Ge and Wang, 2021). Since > 95% of GluA2 are edited (Puchalski et al., 1994), AMPARs that lack GluA2 have been commonly identified by their inward rectification properties and their sensitivity to polyamine or specific antagonists, such as an adamantane derivative IEM-1460 (Schlesinger et al., 2005) or a spider toxin analog 1-naphthylacetyl spermine [NASPM, (Koike et al., 1997)].

Finally, plastic changes in AMPARs are crucial for experience-dependent synaptic plasticity. The most common forms of synaptic plasticity, including long-term potentiation (LTP) and long-term depression (LTD), are cellular mechanisms for learning and memory (Bliss and Collingridge, 1993; Martin et al., 2000; Malenka and Bear, 2004). AMPAR trafficking is one of the most important mechanisms for the expression of synaptic plasticity (Diering and Huganir, 2018). LTP has been shown to be related to the insertion of GluA2-lacking AMPARs (Guire et al., 2008; Sanderson et al., 2016; Purkey et al., 2018; Ge et al., 2019), followed by the replacement of these receptors with GluA2-containing receptors (Plant et al., 2006; Lu et al., 2007). For instance, the impact of cocaine on enhancing AMPAR function is related to the insertion of GluA2-lacking AMPARs (Bellone and Luscher, 2006; Argilli et al., 2008). On the other hand, LTD is mediated by the endocytosis of AMPARs (Collingridge et al., 2010). Trafficking of AMPARs is closely regulated by glycosylation (Kanno et al., 2010), nitrosylation (Diering and Huganir, 2018), palmitoylation (Sohn and Park, 2019), ubiquitination (Widagdo et al., 2017), and phosphorylation (Diering et al., 2016) of different residues in AMPAR subunits. For instance, the phosphorylation of S880 and tyrosine residues at the end of the carboxyl tail of GluA2 has been related to endocytosis and LTD regulation (Seidenman et al., 2003; Ahmadian et al., 2004).

Apart from Hebbian plasticity, regulation of AMPAR trafficking is important for the expression of homeostatic plasticity (Shepherd and Huganir, 2007). As a negative feedback mechanism, homeostatic plasticity acts to maintain the stable balance of neuronal activities via the internalization and externalization of AMPARs at the postsynaptic surface. For instance, calcium-permeable GluA2-lacking AMAPRs have been implicated in the homeostatic scaling-up in response to inactivation (Turrigiano, 2007). In response to overactivation, AMPARs are removed from the postsynaptic surface through endocytosis and protein degradation (Hou et al., 2011).

The contribution of AMPAR molecular composition, function, and plastic properties in social behaviors varies. We will focus on aggression, sociability, and social memory which have been extensively examined in rodent studies. Since rodent models have been used for examining social deficits in brain diseases, including ASD and schizophrenia, we will review the contribution of AMPARs to social deficits in these models.

## 3 Role of AMPARs in naturalistic social behaviors

### 3.1 Aggression

#### 3.1.1 GluA1

**FIGURE 2:**
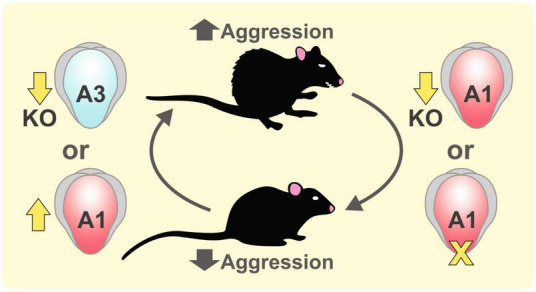
Role of GluA1 and GluA3 subunits in aggression. Aggressive behaviors in mice can be increased by knocking out GluA3 subunit (Adamczyk et al., 2012) or by enhancing the expression of GluA1 subunit by the treatment of a ketamine metabolite (2R,6R)-hydroxynorketamine (Ye et al., 2019). Reducing GluA1-mediated AMPAR function in GluA1 knockout mice and GluA1 knockin mice with a channel pore mutation suppressed aggressive behaviors (Vekovischeva et al., 2001).

Aggression refers to attacks on another individual with an intent to cause harm. In mice, aggression occurs in social conflicts to defend intruders or to protect their pups (Miczek et al., 2001). While aggressive behaviors are important for establishing social hierarchy and gaining reproductive success, aggression is associated with various neurological and psychiatric disorders (Lane et al., 2011). In the search for genetic factors for aggression, findings from knockout (KO) models or inbred strains with high aggressiveness have shown that the expression levels of various AMPAR subunits are related to the expression of aggression (Figure 2). Vekovischeva et al. found that GluA1 KO mice or mice with a knockin (KI) mutation replacing the Q codon with an R in the GluA1 channel pore, which decreased AMPAR function (Vekovischeva et al., 2001), showed reduced aggressive behaviors and increased exploration time to the opponent and environment (Vekovischeva et al., 2004). The resident-intruder test showed that GluA1 KO mice exhibited significantly lower aggression compared to wildtype (WT) mice. The same group further investigated the role of AMPARs in aggression by intraperitoneal injection of AMPAR antagonists to aggressive Turku mice, an aggressive strain from selective breeding, and found that aggressive behaviors were reduced (Vekovischeva et al., 2007). Competitive antagonists NBQX and CBQX reduced only the biting behavior of Turku mice, while non-competitive antagonist GYKI eliminated all aggression behaviors. The stronger behavioral effect of GYKI further supports the role of AMPARs in mediating aggressive behaviors. In addition, treating rats with (2R,6R)-hydroxynorketamine, a metabolite of ketamine that augmented glutamatergic transmission, not only increased AMPAR-mediated miniature excitatory postsynaptic currents (mEPSCs, Ye et al. (2019)) and GluA1 expression (Chou et al., 2018) in the ventrolateral periaqueductal gray, but also elevated aggressive behaviors including biting and threatening.

Testosterone is a hormonal factor mediating male aggressive behavior. For instance, early testosterone administration to neonatal mice can enhance their aggressive behavior (Edwards, 1969). It has been shown that testosterone could alter aggressive behaviors via changing AMPAR functions. Injecting adolescent hamsters repeatedly with anabolic/androgenic steroids (a mixture of different forms of testosterones) enhanced the aggressive behaviors of male hamsters. These steroids not only increased the expression of phosphate-activated glutaminase, a rate-limiting enzyme for glutamate synthesis, but also increased GluA1 expression in the ventrolateral hypothalamus and bed nucleus of the stria terminalis, areas of the brain involved in aggressive behaviors (Fischer et al., 2007). Androgen receptors in the hypothalamus and limbic system also colocalize with AMPARs, suggesting androgen exposure could elevate aggressive behaviors by altering AMPAR activities.

#### 3.1.2 GluA2

Much less is known about the role of GluA2 in aggression. Since aggression is regulated by the serotonergic system, tryptophan hydroxylase-2 (TPH2), a rate-limiting enzyme in the synthesis of serotonin, has a significant impact on aggressive behaviors in stressed mice. Heterozygous TPH2(+/-) male mice were phenotypically normal but became aggressive after predation stress and exhibited a higher expression of GluA2 in the PFC, hippocampus, and striatum compared to non-stressed controls (Gorlova et al., 2020). However, the expression levels of other AMPAR subunits were not compared in this study. In addition, this increase in GluA2 was not found in female TPH2(+/-) mice after stress (Svirin et al., 2022).

#### 3.1.3 GluA3

Comparing different inbred mouse strains revealed some extremely aggressive strains like NZB/B1NJ mice and non-aggressive strain A/J mice. Using resident-intruder tests, Brodkin et al. (2002) identified two quantitative trait loci between the aggressive NZB/B1Nj and non-aggressive A/J strains in the X chromosome, which contains the candidate gene gria3 for the GluA3 subunit of AMPARs. Interestingly, gria3 was also implicated in human aggression. Missense mutations or expression-reducing single nucleotide polymorphisms of gria3 were identified in patients with aggressive outbursts or violent criminal history (Peng et al., 2022). To examine the causal relationship between GluA3 expression levels and aggression, they examined the effect of manipulating AMPAR function in GluA3 KO mice. By knocking out GluA3, Adamczyk et al. (2012) found increased aggressive behaviors in GluA3 KO mice compared to controls. GluA3 KO male mice also exhibited enhanced dyadic male-male nonaggressive behaviors than controls in a neutral environment. Peng et al. (2022) found that compared to the control, GluA3 KO mice exhibited lower mEPSC frequency and amplitude in the PFC. Aggressive behaviors were reduced by overexpressing GluA3 in the PFC of GluA3 KO mice.

#### 3.1.4 Auxiliary subunits

Finally, knocking down the auxiliary subunit of AMPARs can also induce aggression. Through genetic screening in anti-social personality disorder patients, Peng et al. (2021) identified a single nucleotide polymorphism rs10420324G on TARP γ8, which reduces TARP γ8 expression. TARP γ8 KO mice exhibit antisocial personality disorder-related behaviors like aggression, impulsivity, and risk ignoring. Taken together, while aggression can be promoted by GluA1 expression, increasing the expression of GluA3 and TARP γ8 could suppress aggressive behaviors.

### 3.2 Sociability and social memory

Social behaviors are commonly examined using two behavioral tasks, namely sociability and social novelty preference. Sociability refers to the preference of interacting with a social target when compared to an inanimate object. Social interaction for rodents is generally rewarding and social play can be commonly observed in rodents (Vanderschuren et al., 2016). The preference for a social vs. non-social object is typically examined in a 3-chamber apparatus. In addition, because of the propensity to interact with a novel social target, social novelty recognition has been used to study social memory. Either using a 3-chamber test (Yang et al., 2011) or a habituation-dishabituation task (Gheusi et al., 1994), the reduction of interaction time with the familiar mouse indicates the formation of social memory. As we discuss below, various studies have revealed the contribution of AMPAR subunits to rodent performance in the sociability and social novelty tasks.

#### 3.2.1 GluA1

GluA1 KO mice displayed reduced sociability and disorganized social behaviors, including shorter individual investigative bouts of an unfamiliar conspecific and more non-anogenital investigations than WT mice (Wiedholz et al., 2008). Kilonzo and coworkers used transgenic models to specifically knock down GluA1 in different hippocampal regions (Kilonzo et al., 2022). They found that knocking down GluA1 in the CA2 reduced reciprocal social interaction between conspecifics and impaired short-term (but not long-term) social memory. However, knocking down GluA1 in the CA3 improved social interaction and impulse control.

The integrity of social memory has been related to the stability of GluA1 mRNA. FUS, a DNA/RNA binding protein, has been associated with amyotrophic lateral sclerosis and frontotemporal dementia (FTD) that present not only prominent degeneration of motor neurons but also deficits in social behaviors. Udagawa et al found that FUS binds the 3′end of GluA1 mRNA and contributes to its stability (Udagawa et al., 2015). Knocking down FUS in the mouse CA1 region, not only decreased GluA1 expression and excitatory synaptic currents, but also induced anxiety, hyperactivity, and impaired social memory for a familiar mouse.

Apart from the hippocampus and cortex, the impact of GluA1 in other brain regions on social behaviors has also been examined. In mice lacking Arc, an immediate early gene and activity-regulated cytoskeleton-associated protein, the surface expression of GluA1 is enhanced in the nucleus accumbens (NAc) but not in the PFC, dorsal hippocampus, and amygdala (Penrod et al., 2019). Arc KO mice also displayed deficits in social novelty preference, which was rescued by NAc Arc overexpression. Finally, impaired social novelty preference was observed by knocking down Arc in the NAc alone.

#### 3.2.2 GluA2

Although GluA2 KO mice exhibited changes in synaptic plasticity and cognitive functions, no studies have examined social behaviors in those mice. Nonetheless, regional-specific changes in the GluA2 subunit have been observed in animal models with social impairment. Mice expressing mutated multivesicular body protein 2B (CHMP2B), an FTD-associated gene, exhibited an age-dependent impairment in sociability and other neurodegenerative phenotypes (Gascon et al., 2014). These mice also showed enhanced expression of GluA2, GluA3, and GluA4 subunits in the postsynaptic density (PSD) of the PFC. Enhanced AMPAR function associated with these changes was likely related to the social deficits in these mice, since blocking AMPAR by NBQX increased their sociability.

A decrease in GluA2 in the hippocampus was also associated with social impairment. CDKL5 (cyclin-dependent kinase-like 5) deficiency disorder is a rare disease associated with intellectual disability and impaired sociability. CDKL5 has been shown to regulate GluA2 expression (Yennawar et al., 2019). Transgenic mice that carry a mutation in CDKL5 (R59X) exhibited lower synaptic expression of GluA2 in the hippocampus. These mice also showed impaired social novelty preference. In a model of Alzheimer’s disease (AD), 3xTg-AD mice which express AD-related mutations of the amyloid precursor protein, presenilin 1 and tau, hippocampal GluA2 surface expression was decreased. 3xTg-AD mice also developed cognitive impairment and social deficits. The levels of collapsin response mediator protein 5 (CRMP5), a member of CRMPs that play important roles in brain development and spinogenesis, were significantly increased in the hippocampus of 3xTg-AD mice (Lin et al., 2019). Knocking down CRMP5 with shRNA not only rescued the social deficits but also reduced the level of S880 phosphorylation and the level of surface GluA2. Notably, increased hippocampal GluA2 expression was observed in an animal model of long-term allergic airway inflammation that exhibited impaired sociability (Saitoh et al., 2021). These findings may suggest that too high or too low GluA2 expression could be detrimental to social cognition.

Since the GluA2 subunit determines the calcium permeability and gating properties of AMPARs, functional roles of GluA2-containing and -lacking receptors on social behaviors have been examined. In CHMP2B mutant mice, the overall amplitude and frequency of AMPAR-mediated mEPSCs are unchanged, but the calcium-permeable, GluA2-lacking AMPAR is largely replaced by calcium-impermeable AMPAR due to the increase in GluA2 (Gascon et al., 2014). miR-124, a regulator of the expression of AMPAR subunits, is reduced in CHMP2B mutant mice, thus possibly responsible for the alteration of AMPAR subunits. Overexpression of miR-124 or knocking down GluA2 expression in the PFC of these mice rescued social deficits. On the other hand, decreased expression of GluA2 in CDKL5 (R59X) mice resulted in an increased proportion of GluA2-lacking AMPAR, increased inward rectification of excitatory postsynaptic currents, and impaired LTP (Yennawar et al., 2019). GluA2-lacking AMPAR antagonists IEM-1460 and NASPM rescued the sociability of R59X mice, further supporting a causal relationship between GluA2-lacking AMPAR function and social behaviors.

**FIGURE 3:**
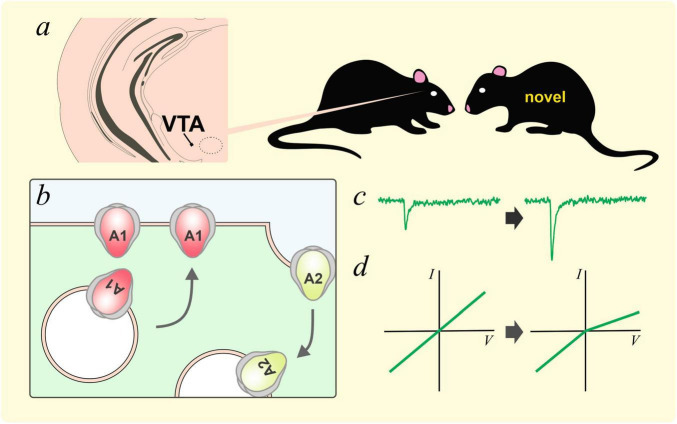
GluA2 lacking AMPARs and social novelty. **(a)** Interaction with a novel social target has been shown to increase AMPAR-mediated currents in dopaminergic neurons in the ventral tegmental area (VTA, Bariselli et al., 2018). This plastic process could be caused by the removal of GluA2-containing AMPARs and the insertion of GluA2-lacking AMPARs, likely homomeric GluA1 AMPARs **(b)**. These plastic changes resulted in an increase in AMPAR currents **(c)** and inward rectification **(d)**.

GluA2 trafficking in the ventral tegmental area (VTA) is an important plastic process for the recognition of social novelty (Figure 3). Bariselli et al. (2018) used chemogenetics to show that the exploration of novel social targets, but not novel objects, was enhanced by the activity of VTA dopaminergic neurons. The exposure to a novel social target was associated with the insertion of a GluA2-lacking subunit and enhanced rectification of AMPAR current in the VTA. Inhibiting GluA2-lacking AMPARs with NASPM enhanced the habituation effect of familiar conspecifics. On the other hand, activating GluA2-lacking AMPARs with optogenetics inhibited the habituation process. Interestingly, knocking down neuroligin3 in VTA dopaminergic neurons, which impaired social novelty preference and sociability, resulted in an aberrant overexpression of GluA2-lacking AMPARs and occluded novel conspecific induced plasticity. This group also showed that a week of social isolation during adolescence resulted in increased social interaction (Musardo et al., 2022). However, socially isolated mice exhibited impaired social habituation and social novelty preference, suggesting an impairment of social memory. These behavioral changes are related to the activation of oxytocin neurons in the paraventricular nucleus of the hypothalamus (PVH), and the removal of GluA2 in AMPARs within the VTA. Notably, blocking calcium permeable GluA2-lacking AMPARs was sufficient to inhibit the increase in social interaction caused by social isolation. Taken together, these findings support the important roles of GluA2-lacking AMPARs in VTA dopaminergic neurons in social novelty detection and social cognition.

#### 3.2.3 GluA3

Reducing GluA3 expression enhanced aggression, sociability, and dyadic interaction between male mice (Adamczyk et al., 2012). However, when GluA3 KO and WT mice were under a high-fat diet, only GluA3 KO mice displayed impairments in sociability and social novelty preference (Li et al., 2023). Autoantibodies against AMPARs have been associated with disorders with social impairment, such as FTD. Autoantibodies against the GluA3 subunit have been identified in FTD patients. Mice that received intracerebroventricular injection of these antibodies not only displayed reduced GluA3 expression in PSD and altered spine morphology in the PFC (not in the hippocampus) but also showed impairment in sociability and poorer discrimination of the emotional states of conspecifics (Scheggia et al., 2021). This suggested an impairment in social preference and the recognition of social valence, the emotional valence associated with a social target. Notably, GluA3 autoantibodies did not affect the total expression level of GluA3, suggesting an effect of these antibodies on the trafficking of GluA3.

#### 3.2.4 Auxiliary subunits

Pro-social behaviors were also affected by the auxiliary subunit TARP. Knocking in TARP γ2 with the V143L mutation resulted in impaired AMPAR functions in the hippocampus. In addition, these mice showed deficits in social novelty preference (Caldeira et al., 2022). Social deficits were found in homozygous, but not heterozygous, KI mice. Although the total expression of GluA1 and GluA2 was not affected in mutant mice, coimmunoprecipitation studies revealed a lower interaction between GluA1 and TARP γ2, and the surface expression and synaptic level of GluA1 were reduced.

#### 3.2.5 Plasticity of AMPARs

The role of AMPAR trafficking in regulating social behaviors suggests that social behaviors are regulated by the plastic properties of AMPAR. N-ethylmaleimide-sensitive factor (NSF) is a known player that mediates AMPAR endocytosis. Xie et al have shown that heterozygous NSF(+/-) mice displayed reduced expression of NSF, lower density of AMPAR in the PSD, and reduced LTD (Xie et al., 2021). NSF(+/-) mice also exhibited impaired sociability and social vocalization.

Prex1 is a Rac-specific Rho GTPase guanine nucleotide exchange factor. Synaptic P-Rex1 signaling regulates hippocampal LTD and autistic-like behavior. Copy number deletion of Prex1 has been associated with ASD. Mice with a congenital KO or hippocampal knockdown (KD) with shRNA of Prex1 displayed normal spatial and fear memory formation, but impaired reversal spatial learning, fear extinction, and social novelty recognition (Li et al., 2015). Prex1 KO mice displayed normal basal synaptic transmission and LTP formation. However, NMDAR receptor-dependent LTD and AMPAR endocytosis were impaired in Prex1 KO mice. Since Prex1 is a substrate of protein phosphatase 1α (PP1α), which mediates AMPAR endocytosis, these findings support the role of Prex1 as a downstream signal for PP1α to regulate social behaviors.

GRIP1/2 is a well-characterized regulator of AMPAR recycling. Since the gain of function of GRIP1/2 has been associated with ASD, Han et al tested the effect of double KO of GRIP1/2 on sociability and social novelty preference (Han et al., 2017). In GRIP1/2 KO mice, GluA2 S880 phosphorylation was increased in the frontal cortex and cerebellum. They also showed that both the sociability and social novelty preference of GRIP1/2 KO mice were higher than WT controls. Since only the total expression of GluA2 subunits was examined, the impact of GRIP1/2 KO on other AMPAR subunits and the functional properties of these receptors remains unclear.

Apart from deficits in AMPAR endocytosis and LTD, LTP deficit has also been implicated in social impairment. Mice with the expression of dominant negative retinoic acid receptor exhibited poorer social memory, reduced hippocampal AMPAR-mediated synaptic transmission and LTP (Nomoto et al., 2012). Li et al showed that GluN1 S897A mutant mice had disrupted S897 phosphorylation and reduced synaptic incorporation of GluN1. These mice displayed lower NMDAR and AMPAR transmission, reduced GluA1 expression in the hippocampal CA1, and a disruption in LTP (Li et al., 2009). These mice also performed poorly in a 5-trial habituation-dishabituation task, supporting a link between LTP dysfunction and social impairment.

## 4 Roles of AMPARs in brain disease models

### 4.1 Autism spectrum disorders

Autism spectrum disorder (ASD) is a severe neurodevelopmental syndrome that includes a range of disorders including autistic disorder, pervasive developmental disorder, and Asperger syndrome. ASD is characterized by a common deficit in social communication. It affects about 1 in 36 children in the United States (Maenner et al., 2023) and has increased prevalence over the years. Currently, the mechanism underlying social impairment in ASD is not well understood, and no effective treatment has been developed to relieve the core social symptoms of ASD. Thus, using animal models to study social deficits could elucidate future therapeutic targets to treat ASD.

#### 4.1.1 Shank-related ASD models

SH3 and multiple ankyrin repeat domains protein (Shank) is a family of scaffolding proteins in excitatory synapses (Monteiro and Feng, 2017). Various binding domains of Shank allow it to interact with ions channels (e.g. AMPARs), cytoskeletons, and other signaling proteins in the PSD. There are 3 members in the Shank protein family: Shank1, Shank2, and Shank3. There are also multiple isoforms of each Shank protein through splicing sites and epigenetic controls. Although possessing similar features, expressions of Shank1, Shank2, and Shank3 are region-specific. Shank1 is enriched in the cortex, thalamus, and hippocampal CA1 and CA3. While Shank2 can be found in these brain areas, it also expresses in the kidney. Apart from expressing widely in the brain, Shank3 is the only one that is enriched in corticostriatal glutamatergic synapses. Different isoforms of Shank3 are also expressed in the brain in a region-specific manner.

Through its PDZ domain, Shank has been shown to regulate the expression and function of AMPARs. For instance, Shank3 directly interacts with GluA1, but not GluA2, subunits (Uchino et al., 2006). Shank2 and Shank3 are crucial for zinc-dependent AMPAR-mediated neurotransmission during development by facilitating the replacement of GluA1 with GluA2 in synapses (Ha et al., 2018). Shank3-Rich2 interaction has also been shown to regulate the trafficking of GluA1 for AMPAR recycling and LTP (Raynaud et al., 2013).

Shank mutations were identified in ASD patients. Mice with mutations in Shank display behavioral abnormalities resembling symptoms in ASD patients, including social impairment and repetitive behaviors. Below we summarize changes in AMPAR caused by ASD-related Shank mutations and the contribution of AMPAR to the social deficits of these models.

##### 4.1.1.1 Shank3

Among the Shank family, Shank3 is the most investigated. Shank 3 is located on chromosome 22q13 in humans, and the loss of one copy of Shank3 leads to chromosome 22q13 deletion syndrome [22q13DS, also called Phelan-McDermid syndrome, (Phelan and McDermid, 2012)], which is a cause of ASD. Other mutations, deletion, translocation, and ring chromosome of Shank3 can also lead to ASD with differing severity. Modeling ASD by mutating Shank3 has been well-established.

Targeted deletion of Shank3 exons 4-9 encoding ankyrin repeat domain produced haploinsufficiency. Mutant male mice exhibited reduced social sniffing and lower female mice-related ultrasonic vocalization, indicating social impairment (Bozdagi et al., 2010). Lower mEPSC functions, GluA1-labeled puncta, impaired LTP, but intact LTD, were found in the hippocampus of mutant mice. Expansion of spines after induced LTP was also transient in mutant mice compared to the consistent spine size expansion in WT mice. Another study showed that apart from displaying a reduced expression of GluA1 and spine density in the hippocampal CA1 region, these mutant mice exhibited impaired sociability and ultrasonic communication (Wang et al., 2011). Peca et al. also showed impaired social interaction and social novelty recognition in mice with Shank3 haploinsufficiency (Peca et al., 2011). Expression of GluA2, GluN2A, and GluN2B was decreased in the striatum. Mutant mice also displayed lower mEPSC frequency and amplitudes than WT in medium spiny neurons. While these studies only examined the expression of 1 of the 3 isoforms of Shank3, deleting exon 21 including the Homer binding domain resulted in the loss of all Shank3 isoforms (Kouser et al., 2013). Mice that lack all 3 Shank3 isoforms (Shank3 KO mice) exhibited impairment of social novelty preference and increased grooming. Interestingly, AMPAR subunit expression in hippocampal synaptosomes was not altered, while mGluR5 (metabotropic glutamate receptor subtype 5) levels were increased few-fold. Functionally, the input/output relationship of evoked excitatory postsynaptic potentials (EPSP) and mEPSC frequency was reduced in these KO mice, suggesting a reduction in hippocampal AMPAR function.

Knocking out Shank3 in the anterior cingulate cortex (ACC) alone triggered ASD-related social impairments (Guo B. et al., 2019). ACC Shank3 KO mice exhibited reduced spine density and PSD length when compared to WT. These structural changes were associated with reduced AMPAR-mediated mEPSCs and LTP deficits. ACC Shank3 KO mice also showed deficits in sociability and social novelty preference, while other behaviors including grooming and anxiety were untouched. Accordingly, ACC pyramidal neurons were hypoactive in these mice. Optogenetic activation or re-expressing Shank3 in the ACC improved social interaction. In addition, enhancing AMPAR function by ampakine, a positive allosteric modulator (PAM), was sufficient to rescue social impairment in ACC Shank3 KO mice, supporting a causal relationship between AMPAR dysfunction in the ACC and social impairment.

Epigenetic mechanisms that result in social impairment in ASD and the Shank3 haploinsufficiency ASD model could lead to changes in AMPAR function. Rapanelli et al. (2022) found that PFC levels of H3K4me2, an epigenetic marker on histones that regulates transcription, were reduced in ASD patient postmortem tissue and in mice with Shank3 haploinsufficiency. They also found lower AMPAR-mediated mEPSC functions in mutant mice. An inhibitor of histone demethylase 1, GSK-LSD1, not only elevated H3K4me2 level, but also rescued social deficits and NMDAR and AMPAR hypofunction in the PFC and striatum of mice with Shank3 haploinsufficiency, respectively.

##### 4.1.1.2 Shank2

Although far less common than Shank3-related mutations, duplication/triplication of the Shank2 locus has been identified in some ASD patients. Shank2 KO mice displayed deficits in sociability and social novelty preference, and a decrease in GluA1 expression in the hippocampus (Peter et al., 2016). Eltokhi et al. (2021) have shown that using a forebrain doxycycline-controlled Tet-Off system, overexpressing Shank2A(WT) or Shank2A(R462X), an extrasynaptic mutant, displayed different alterations in hippocampal AMPAR function: Shank2(WT) overexpression caused reduced GluA1 and GluA2 levels, and an increase in calcium-permeable AMPARs; Shank2(R462X) mice lacked these synaptic changes. In Shank2(WT) mutant mice, enhancement of the high conductance calcium-permeable AMPAR subtype is specific to apical dendrites, evidenced by reduced NASPM-sensitive currents and impaired LTP in basal dendrites but increased NASPM sensitivity in apical dendrites. Shank2(WT) mice exhibited impaired social behaviors, such as reduced sociability and nesting behavior abnormality. However, Shank2A(R462X) mice exhibited behaviors resembling attention deficit hyperactivity disorder, namely enhanced novel object recognition, vocalization, grooming, and sociability. These findings suggest that social behaviors are regulated by subcellular changes in AMPAR function in hippocampal neurons. Interestingly, the impact of Shank2(WT) or Shank2(R462X) overexpression on social behaviors can be attenuated by turning off their expression with doxycycline, supporting a causal relationship.

##### 4.1.1.3 Shank1

Social deficits and AMPAR dysfunction in ASD have been associated with mutations in Shank1. Qin et al. (2022) used CRISPR-Cas9 to create a KI missense mutation, R882H-KI, that imitated a c.2621 G > A mutation of Shank1 in ASD patients. Mutant mice displayed reduced spine density, impaired hippocampal LTP, and social novelty preference. These changes likely resulted from a disruption in the signaling pathway mGluR1-IP3R1-calcium, a master regulator of glutamatergic synapses. Another ASD-related mutation model, Shank1 P1812L KI mice (Qin et al., 2023), also exhibited decreased mGluR1-IP3R1-calcium signaling and similar behavioral phenotypes as the R882H-KI mice. Interestingly, homozygous, but not heterozygous, P1812L KI mice were found to have increased GluA2 expression. While these studies did not reveal an association between AMPAR expression levels and social behaviors, the importance of mGluR1 in regulating AMPAR functions (Bellone and Luscher, 2005) calls for more studies investigating AMPAR functional changes and their relationship with social impairments in these Shank1 models.

#### 4.1.2 Valproic acid-induced model of ASD

Valproic acid (VPA) is a well-known chemical that increases the susceptibility to ASD under prenatal exposure (Nicolini and Fahnestock, 2018). Treating pregnant rats with VPA could induce ASD-related behaviors, such as social impairment in offspring. Various studies have revealed the contribution of AMPAR changes to social impairment in the VPA model.

In the lateral amygdala of VPA-exposed offspring, AMPAR-mediated mEPSC frequency and amplitude were enhanced (Lin et al., 2013), a presynaptic form of LTP that resulted in the hyperactivity of the lateral amygdala. These synaptic and cellular changes were thought to be the cause of impaired social interaction, increased anxiety, and abnormal fear conditioning. Wu et al. (2018) have shown a similar effect of VPA in enhancing amygdala AMPAR activity and ASD-like behavior. Intra-amygdalar injection of D-cycloserine (DCS), an NMDAR partial agonist, relieved social impairment by inducing NMDAR-mediated LTD and facilitating the recycling of GluA2 in the amygdala. Blockade of AMPAR endocytosis by targeting the GluA2 subunit using Tat-GluA2-3Y peptide abolished the effect of DCS on rescuing social impairment, suggesting a causal relationship between GluA2 enhancement in the amygdala and social impairment in VPA-exposed offspring. This group further showed that VPA-exposed offspring exhibited impaired endocannabinoid-mediated LTD (Wu et al., 2020). URB597, an inhibitor of endocannabinoid (eCB) anandamide, rescued both the eCB-mediated plasticity and sociability by facilitating AMPAR endocytosis. Similarly, this effect was abolished by blocking GluA2 endocytosis with the Tat-GluA2-3Y peptide, further demonstrating that enhanced GluA2 surface expression caused social impairment. In other studies, reducing AMPAR expression in the hippocampus and PFC with an extract of Bacopa monnieri, an herbal medicine found in northeast India (Abhishek et al., 2022), or Granulocyte Colony-Stimulating Factor (Mishra et al., 2022), rescued social novelty recognition deficit in VPA-exposed offspring.

VPA has also been suggested to cause social impairment and synaptic pathology through an imbalance between excitatory and inhibitory synaptic transmission resulting from oxidative stress. Schiavi et al. (2022) examined the behavioral and synaptic effect of an antioxidant, N-acetylcysteine (NAC), on VPA-exposed offspring. NAC not only rescued sociability in VPA-exposed offspring and brain glutathione level but also VPA-induced changes in the mRNA expression of GluA1 and GluA2 in the hippocampus, NAc, and cerebellum. Indeed, a meta-analysis recently showed positive effects of NAC on ASD patients (Lee et al., 2021). Further investigation of the effect of NAC on social behaviors could provide a better understanding of the molecular mechanisms underlying AMPAR subunits and social impairment in ASD.

Changes in AMPARs after prenatal VPA exposure have also been shown to alter inhibitory neuronal function. Wang et al. (2018) showed that VPA exposure reduced neuronal nitric oxide synthase (nNOS) levels in offspring, including the number of nNOS-positive GABAergic interneurons in the mouse basolateral amygdala (BLA). They also found a similar loss of nNOS interneurons in the BTBR ASD model, suggesting an imbalance between excitatory and inhibitory synaptic transmission in ASD. By characterizing membrane proteins of nNOS interneurons using label-free liquid chromatography–mass spectrometry analysis and western blot, the expression of GluA4 was found to be significantly decreased in the BLA of mice that received early postnatal treatment of VPA (Wang et al., 2021). GluA4 is predominately expressed early in development and has limited expression in the adult brain, suggesting it is a molecular culprit for the abnormal development of nNOS interneurons in the BLA. Changes in GluA4 were also revealed in an epigenetic study investigating differentially expressed miRNAs (Chen et al., 2023), which also found changes in miRNA pathways that are involved in LTD and GluA2 expression in VPA-exposed offspring.

#### 4.1.3 Other ASD-related transgenic models

Apart from the Shank and VPA models, changes in AMPARs have been examined in various genetic models of ASD that exhibit social deficits. For instance, reduced AMPAR function and spine density were found in the cortex of mice lacking NOMA-GAP (Schuster et al., 2015), a Cdc42 GTPase-activating multiadaptor protein, and in the hippocampus of mice lacking Lrfn2 (Morimura et al., 2017), a transmembrane protein that binds with PSD95 through a PDZ domain. In addition, knocking down glypican 4, a hippocampally expressed heparan sulfate proteoglycan that is crucial for clustering GluA1-containing AMPARs in synapses, resulted in reduced GluA1 expression (Dowling and Allen, 2018). These mice also showed hyperactivity at the juvenile state and impairment in sociability and social novelty recognition in adulthood. These alterations in behavior are consistent with the alterations in GluA1 KO mice (Maksimovic et al., 2014).

Increased AMPAR expression and function have also been shown in ASD-related mouse models. For instance, mutation of the MAM domain containing glycosylphosphatidylinositol anchor 2 (MDGL2) has been associated with ASD. Connor et al. (2016) showed that haploinsufficiency of MDGL2 enhanced hippocampal AMPAR function and impaired LTP. These mice also exhibited ASD-related symptoms, such as reduced social interaction and sociability. EPAC2, a guanine nucleotide exchange factor that regulates Rap and Ras, colocalizes with AMPAR subunit GluA2/3 and facilitates the removal of GluA2/3 from synapses. Based on a rare variant of EPAC2 in ASD patients, mice lacking EPAC2 were generated. These mice displayed reduced sociability and male-female ultrasonic vocalization, consistent with autistic-like behaviors (Srivastava et al., 2012). Comparing synaptic properties of cortical cultures derived from EPAC2 KO mice revealed larger spines and higher GluA2/3 expression (Jones et al., 2019). In addition, knocking out EPAC2 also increased the expression of vesicular transporters for GABA, but not glutamate, suggesting an imbalance in excitation/inhibition toward inhibition. Taken together, these findings suggest that too little or too much AMPAR function could lead to social impairments in these animal models.

While the previously described findings are correlational, causal relationships between AMPAR changes and social deficits have been examined. ASD-related social impairment can be induced by knocking out vaccinia-related kinase 3 (VRK3), a type of casein kinase. Although the mechanisms of VRK3 are not well understood, VRK3 KO mice displayed impaired sociability and social novelty (Kang et al., 2017). They also demonstrated higher grooming, lower pup retrieval and nesting behaviors, lower anxiety, and impaired performance in passive avoidance, novel object recognition, and Barnes maze. VRK3 KO mice exhibited reduced PSD thickness and length. Although no changes in AMPAR subunit expression were found, these mice showed a reduction in calcium-permeable AMPAR currents. In addition, expression levels of other synaptic proteins such as Arc, PSD-95, and TrkB were altered. Interestingly, the alteration in synaptic protein profile and social deficits were partially rescued by 7,8-dihydroxyflavone (7,8-DHF), an agonist of TrkB. IQ motif and Sec7 domain 2 (IQSEC2) is an X-linked gene that is associated with intellectual disability and ASD. Using IQSEC2 KO mice, Mehta et al. (2021) observed significant deficits in sociability and social novelty preference. These mice exhibited reduced mEPSC and miniature inhibitory postsynaptic current function in the PFC. Both AMPAR- and NMDAR-mediated currents were reduced in the KO mice. These synaptic changes and social deficits were rescued by overexpressing IQSEC2.

Social deficits in ASD mouse models have also been rescued by directly modulating AMPAR functions. Sacai et al. (2020) examined the impact of contactin-associated protein-like 2 genes (CNTNAP2) and Abelson helper integration site-1, two ASD-related mutated genes, specifically in layer 2/3 pyramidal neurons in the PFC. Separately knocking down these two genes in layer 2/3 pyramidal neurons resulted in mice with impaired sociability and reduced AMPAR-mediated synaptic transmission. Impaired social behaviors of both knockdown mice were rescued by enhancing excitatory synaptic transmission with CX546, a PAM of AMPARs. In addition, enhancing AMPAR function with different forms of ampakine increased the sniffing time of BTBR Tþtf/J mice, which is a model of ASD with social impairments (Silverman et al., 2013). Notably, the loss of preference of a social target vs. an inanimate object in BTBR mice was not rescued by ampakine. While most studies examined social behaviors between same-sex dyads, some mutations have specifically affected opposite-sex interactions. Jabarin et al. (2021) showed that preference for the opposite sex was impaired in mice with the A350V mutated IQSEC2 gene. In addition, the impact of social isolation on enhancing social interaction was impaired in these mutant mice. Finally, an AMPAR PAM PF-4778574 rescued these social deficits.

Having a balanced level of AMPAR function is important for normal social behavior as shown by Kim et al. using two ASD models (Kim J. W. et al., 2019). They found that both CNTNAP2 and VPA-induced ASD animal models exhibited impaired sociability and social novelty preference, resembling autistic-like behaviors. CNTNAP2 KO mice showed reduced expression of GluA1, GluA2, GluN2A, and GluN2B and reduced mEPSC amplitudes in the PFC. In contrast, VPA-exposed offspring showed enhanced expression of GluA1 and GluN2B and increased mEPSC amplitudes in the PFC. AMPAR changes in these mice were related to social impairment, which can be rescued in CNTNAP2 KO mice by AMPAR agonist PF4778574, and by blocking AMPARs with CP465022 in VPA-exposed offspring. They also showed that social impairment can be induced by either enhancing or reducing AMPAR function by these drugs in control mice.

Finally, the plastic properties of AMPAR also mediate social deficits in some ASD models. A mouse model of Fragile X Syndrome with a KO of the Fmr1 gene not only resulted in anxiety and abnormal social behaviors but also increased GluA2 surface expression and reduced S880 phosphorylation of GluA2 in the hippocampus (Marsillo et al., 2021), the phosphorylation that is related to AMPAR endocytosis. Enhanced CA1 activity caused by these synaptic changes suppressed PKCε signaling in oxytocinergic neurons in the PVH, leading to social deficits and hyper-anxiety in male KO mice. Activating oxytocinergic PVH neurons with the PKCε stimulator DCP-LA not only facilitated GluA2 S880 phosphorylation and reduced the AMPAR surface expression in CA1, but also rescued social impairment in Fmr1 KO male mice.

Directly targeting signaling molecules for AMPAR plasticity could rescue social deficits in ASD models. Guo D. et al. (2019) have shown that global knocking down Dock4, a Rac1 guanine nucleotide exchange factor that is located at the ASD-associated loci 7q31.1, resulted in an impairment in social and object recognition, and spatial learning. Rac1 is also an important player in LTP (Martinez and Tejada-Simon, 2011). Conditional Dock4 KO in hippocampal CA1 reduced Rac1 activity and recapitulated the behavior abnormalities observed in global KO mice. They also showed decreased expression of GluA2, GluN1, GluN2A, and GluN2B subunits, AMPAR- and NMDAR-mediated synaptic transmission, and spine density. Social impairment and synaptic deficits of Dock4 KO mice were rescued by either enhancing Rac1 activity through overexpression or increasing NMDAR function by DCS. Apart from LTP, targeting LTD has also been shown to rescue social deficits. δ-catenin is an ASD-associated gene and a cell adhesion molecule that interacts with N-cadherin. It also binds with AMPAR binding proteins such as GRIP to regulate GluA2 expression. Mice with G34S mutation of δ-catenin exhibited increased GSK3β mediated δ-catenin degradation (Mendez-Vazquez et al., 2023). These mice also showed reduced GluA2, but not GluA1, expression in cortical neurons, which was associated with enhanced and reduced activity of cortical excitatory and inhibitory neurons, respectively. Lithium and the KD of GSK3β rescued social behaviors of G34S δ-catenin KI mice and normalized GluA2 expression and the excitability of cortical excitatory and inhibitory neurons.

### 4.2 Schizophrenia

Schizophrenia is a severe psychiatric disorder that affects 5.1 per 1000 individuals in the United States (Wu et al., 2006). Schizophrenia patients usually present multiple facets of dysfunction including positive symptoms (Ruiz-Castaneda et al., 2022): hallucination, delusion, and disorganized behaviors, negative symptoms (Correll and Schooler, 2020): absence of motivation/interest, and asociality, and cognitive impairment (Bowie and Harvey, 2006): memory and learning deficits. In addition, social cognition is always impaired in schizophrenia patients. Deficits in social cognition were suggested to drive the functional outcomes, including social functioning and independent living ability (Dodell-Feder et al., 2015). Understanding the mechanisms of social deficits in schizophrenia, such as through basic neuroscience studies using animal models, is warranted for developing new treatments for the disease.

Schizophrenia has been associated with AMPAR mutations. For instance, a meta-analysis of whole genomes of > 24,248 schizophrenia patients vs. 97,322 control subjects revealed 10 genes with ultra-rare variants that confer the risk for schizophrenia (Singh et al., 2022), including in GluA3 and GluN2A. Schizophrenia is also associated with duplication of the synaptic scaffolding molecule [S-SCAM, (Zhang et al., 2015)], a postsynaptic scaffolding protein controlling AMPAR trafficking and glutamatergic transmission. Mutant mice carrying extra copies of S-SCAM exhibited increased forebrain synaptic expression of GluA2/3 and enhanced hippocampal input/output field EPSP. Interestingly, Shank, a risk gene of ASD, is also decreased in these mice. Behaviorally, female mice displayed deficits in sociability, while male mice displayed impairment in social novelty recognition. This sex difference could be explained by a greater elevation in synaptic GluA2/3 and field EPSP in the hippocampus of male mutant mice.

Syndapin I, a membrane-shaping protein in the BAR domain superfamily, is another schizophrenia-associated gene. Reduced expression of syndapin I has been shown in the dorsolateral PFC of schizophrenia patients (Pennington et al., 2008). Koch et al. showed that syndapin I KO mice exhibited impaired social novelty preference, hyperactivity towards novel objects, and lower anxiety (Koch et al., 2020). Syndapin I KO mice also have smaller AMPAR boutons, lower mEPSC amplitudes in the hippocampus and PFC, and reduced expression of GluA1 and GluA2 in the PSD. In addition, endocytosis of GluA1 and GluA2 during synaptic plasticity was impaired, suggesting a significant role of AMPAR trafficking and the expression of schizophrenia-related behavioral changes.

The impact of modulating AMPAR function on social behavior deficits in animal models of schizophrenia has also been examined. Neonatal ventral hippocampus lesion (NVHL) has been widely used for modeling developmental changes and behavioral deficits related to schizophrenia. Compared to controls, NVHL rats displayed impaired social novelty preference, social interaction deficit, and hyperlocomotion (Sams-Dodd et al., 1997). NVHL resulted in reduced phosphorylated levels of synaptic proteins including GluA1 (S831), CaMKII (T286), PKCα (S657), and GluN1 (S896) in both the hippocampus CA1 region and PFC. ST101, a cognitive enhancer that targets nicotinic cholinergic receptors and presynaptic glutamate release, rescued behavioral deficits in NVHL rats (Yabuki et al., 2019). Notably, ST101 also restored the phosphorylation levels of synaptic proteins in NVHL rats. In another mouse model of schizophrenia, overexpressing miR-124-3p, which is upregulated in the PFC of schizophrenia and bipolar patients (Namkung et al., 2023; Zurawek and Turecki, 2023), led to social deficits in mice. Mice with PFC overexpression of miR-124-3p showed lower GluA2 levels and higher mEPSC amplitude, which are mediated by GluA2-lacking AMPARs. The direct relationship between the upregulation of GluA2-lacking AMPARs and social behavior abnormality was solidified by the finding that selectively inhibiting GluA2-lacking AMPARs attenuated the social deficits (Yennawar et al., 2019).

### 4.3 Other animal models of social impairment

Targeting AMPARs has been shown to rescue social deficits in other animal models of brain disorders. Social impairment has been observed in metabolic diseases, such as diabetes. Diabetic mice induced by streptozotocin exhibited impaired social novelty preference, but not sociability (Ueda et al., 2021b). These mice displayed higher expressions of neuropeptide Y and GluA1 in the hippocampus. Blocking AMPARs by NBQX rescued social novelty recognition deficits in these mice. A follow-up study from this group showed that direct injection of NBQX into either the ventral CA1 or the BLA were both effective in rescuing impaired social novelty preference in these mice (Ueda et al., 2021a).

Stress-related disorders, such as depression, are commonly associated with abnormal social interaction. AMPAR changes have been associated with depressive disorders. For instance, dysfunction of AMPAR trafficking was implicated in depression pathology (Thompson et al., 2015). The role of AMPAR in depression has been further examined in stress-related animal models. Early life stress from neonatal isolation resulted in enhanced social dominance in adult rats, decreased GluA1 surface expression in spines and reduced mEPSC amplitudes in the PFC (Tada et al., 2016). This disruption in AMPAR function is related to the inactivation of ADF/cofilin and the enhancement of actin stability. By constitutively activating ADF/cofilin, the stability of actins was decreased, the surface expression of GluA1 was increased, and social dominance was reduced. Reduced social behaviors in sociability, novelty detection, and dominance can also be induced by chronic restraint stress (CRS, 21 days, 3 hours daily) in mice (Park et al., 2018). Under CRS, expression of GluA2 and GluA1 was decreased in the PFC. Phosphorylation of S831 and S818 of GluA1 that promote exocytosis to the PSD were also decreased by CRS. Administration of the antidepressant fluoxetine increased the phosphorylation of GluA1 and GluA2 and rescued social impairments. Interestingly, stress-induced reduction of GluA1 synaptic expression exerted opposite effects on social dominance. Apart from using different species in these studies, differences in the timing (neonatal vs. adult), duration (5 days vs. 3 weeks), and the stressors used (isolation vs. restraint) may underlie differences in social dominance.

Social avoidance in the chronic social defeat stress (CSDS) model is commonly regarded as a depression-related behavior marking stress susceptibility (Berton et al., 2006). Conversely, animals exposed to CSDS that do not show deficits in social behavior are termed resilient. In the hippocampus, decreased AMPAR expression and transmission have been reported in CSDS-induced social avoidance. BALB strain mice, a genetic model of depression, show greater susceptibility compared to mice of the B6 strain, which is a model of resilience. Both ventral CA1 infusion of an AMPAR potentiator, CX614, and synaptic insertion of GluA1 through the overexpression of TARP γ8, an AMPAR auxiliary subunit important for recruiting AMPARs to synapses (Rouach et al., 2005), had pro-resilient effects on social behavior in defeated BALB mice (Sakai et al., 2021). Blocking GluA1 synaptic incorporation by overexpressing a dominant negative form of GluA1 diminished social interaction in normally resilient B6 mice after defeat. A similar effect was seen following the pharmacological inhibition of TARP γ8-containing AMPAR. Diminished social behavior with decreased hippocampal GluA1 was also demonstrated through the manipulation of caspase-1, which is involved in AMPAR endocytosis (Li et al., 2018). Defeated mice with a homozygous deletion of caspase-1 had rescued social behavior along with increased GluA1 and GluA2 surface expression in the hippocampus. Overexpression of caspase-1 in the hippocampus resulted in social avoidance after a subthreshold defeat paradigm that is not sufficient to induce social deficits. CSDS in juvenile male mice also elicited social avoidance along with a decrease in hippocampal GluA2 expression and spine density suggesting diminished synaptic transmission (Iniguez et al., 2016). Similarly, impaired glutamatergic transmission in the PFC, as evidenced by decreased expression of GluA1 and GluA2, was observed in susceptible mice (Zhang et al., 2016). KD of EphB2, a tyrosine kinase receptor that mediates GluA1 and GluA2 trafficking in neurons, in the PFC of mice exposed to subthreshold stress not only decreased GluA1 and GluA2 expression but also induced social avoidance.

Dysregulation in the trafficking and expression of the GluA2 subunit, globally and within the hippocampus and NAc, was also associated with impaired social interaction. Ellis et al. studied the GluA2 K882A KI mouse, in which phosphorylation of S880 was disrupted. Mutant mice exhibited increased social avoidance compared to WT after 15 days of social defeat (Ellis et al., 2017). ΔFosB is a transcription factor with pro-resilient effects that are thought to be mediated by its function in regulating GluA2 expression (Vialou et al., 2010). Following CSDS, GluA1 and GluA2 expression levels were increased and decreased, respectively, in the NAc of socially avoidant mice. Inward rectification was significantly higher in susceptible mice and correlated with social avoidance, indicating that the observed maladaptive social behavior may be partially mediated by a greater GluA1:GluA2 ratio. A single infusion of NBQX in the NAc after CSDS increased social interaction and this pro-social effect persisted even 1-week post-infusion. Viral overexpression of GluA2 in the NAc was also able to rescue social behaviors in susceptible mice.

Overall, in animal models of depression with diminished social behavior, the effects on AMPAR neurotransmission are region-dependent. Namely, decreased AMPAR function is observed in the hippocampus and PFC, while an increase is seen in the NAc.

## 5 Discussion

Investigating neural mechanisms of social behaviors has revealed significant contributions of AMPARs to various properties of social behaviors. The integrity of AMPAR-mediated synaptic transmission is important for maintaining normal social functioning. Notably, different AMPAR subunits have been shown to play distinct roles in the regulation of social behaviors (Table 1). In addition, plastic properties of AMPAR-mediated synaptic transmission play crucial roles in the detection and storage of social information. Finally, altered AMPAR expression and function are commonly observed in animal models with social deficits (Table 2). Not only was restoring AMPAR function associated with the rescue of social impairment, but drugs that target AMPARs and auxiliary subunits have also shown promising results in normalizing social functioning. Further studies of the role of AMPAR in social behaviors could reveal novel therapies for treating social impairment that is commonly associated with brain diseases.

**TABLE 1:** Summarizes changes in naturalistic social behaviors in rodent models with the modification of different AMPAR and TARP subunits.

AMPAR subunit	Animal model	Changes in AMPAR subunit	Brain region examined	Changes in social behaviors	References
GluA1	GluA1 KO	decrease		decrease aggression	Vekovischeva et al., 2001, 2004
GluA1	GluA1 KI (Q582R)	decrease		decrease aggression	Vekovischeva et al., 2001, 2004
GluA1	GluA1 KO	decrease		decrease sociability	Wiedholz et al., 2008
GluA1	GluA1 regional KO	decrease	CA2	reduce social interaction and short term social memory	Kilonzo et al., 2022
GluA1	GluA1 regional KO	decrease	CA3	improved social interaction and impulse control	Kilonzo et al., 2022
GluA1	reduce mRNA stability	decrease	CA1	decrease social recognition memory	Udagawa et al., 2015
GluA1	Arc regional KO	decrease	NAc	decrease social recognition memory	Penrod et al., 2019
GluA1	GluN1 S897A mutant mice	decrease	hippocampus	decrease social recognition memory	Li et al., 2009
GluA2	Tph2 (+/-) mice	increase	PFC, hippocampus, striatum	increase aggression	Gorlova et al., 2020
GluA2	CHMP2B mutant mice	increase	PFC	decrease sociability	Gascon et al., 2014
GluA2	CDKL5 mice	decrease	hippocampus	decrease social recognition memory	Yennawar et al., 2019
GluA2	3xTg-AD mice	decrease	hippocampus	decrease social recognition memory	Lin et al., 2019
GluA2	Postnatal allergic airway inflammation	increase	hippocampus	decrease sociability	Saitoh et al., 2021
GluA2	WT	decrease	VTA	social novelty detection	Bariselli et al., 2018
GluA2	WT	decrease	VTA	increase social interaction	Musardo et al., 2022
GluA3	GluA3 KO	decrease		increase aggression	Peng et al., 2022
GluA3	GluA3 KO	decrease		increase aggression	Adamczyk et al., 2012
GluA3	CHMP2B mutant mice	increase	PFC	decrease sociability	Gascon et al., 2014
GluA3	GluA3 KO	decrease		decrease social interaction and sociability	Adamczyk et al., 2012
GluA3	GluA3 KO	decrease		decrease sociability and social recognition memory after high fat diet	Li et al., 2023
GluA3	GluA3 antibodies mediated reduction	decrease	PFC	decrease sociability and social valence	Scheggia et al., 2021
GluA4	CHMP2B mutant mice	increase	PFC	decrease sociability	Gascon et al., 2014
TARP γ2	TARP γ2 KI (V143L)	decrease GluA1 and TARP interaction	hippocampus	decrease social recognition memory	Caldeira et al., 2022
TARP γ8	TARP γ8 KO	decrease mEPSC	PFC	increase aggression	Peng et al., 2021

CDKL5, cyclin-dependent kinase-like 5; CHMP2B, multivesicular body protein 2B; KI, knockin; KO, knockout; NAc, nucleus accumbens; PFC, prefrontal cortex; TARP, transmembrane AMPAR receptor regulatory proteins; Tph2, tryptophan hydroxylase-2; VTA, ventral tegmental area.

**TABLE 2:** Summarizes changes in AMPAR expression or function in animal models of brain disorders.

Brain disorder	Mouse model	AMPAR subunit	Changes in AMPAR	Examined brain region	Changes in social behaviors	References
ASD	AHI1 KO		decrease mEPSC	PFC	decrease sociability	Sacai et al., 2020
ASD	CNTNAP2 KO		decrease mEPSC	PFC	decrease sociability	Sacai et al., 2020
ASD	CNTNAP2 KO	GluA1	decrease	PFC	decrease sociability and social recognition memory	Kim J. W. et al., 2019
ASD	δ-catenin (G34S)	GluA2	decrease	cortex	decrease sociability and social recognition memory	Mendez-Vazquez et al., 2023
ASD	Dock4 KO	GluA2	decrease	hippocampus	decrease sociability and social recognition memory	Guo B. et al., 2019
ASD	EPAC2 KO	GluA2,3	increase	cortex	decrease sociability	Jones et al., 2019
ASD	Fmr1 KO	GluA2	increase	hippocampus	decrease sociability	Marsillo et al., 2021
ASD	Gpc4 KO	GluA1	decrease	hippocampus	decrease sociability and social recognition memory	Dowling and Allen, 2018
ASD	IQSEC2 KO		decrease mEPSC	PFC	decrease sociability and social recognition memory	Mehta et al., 2021
ASD	Lrfn2 KO		decrease mEPSC	hippocampus	decrease social interaction	Morimura et al., 2017
ASD	MDGL2 mutant mice		increase mEPSC	hippocampus	decrease social interaction and sociability	Connor et al., 2016
ASD	NOMA-GAP KO		decrease mEPSC	cortex	decrease sociability and social recognition memory	Schuster et al., 2015
ASD	Shank1 (KD in PV neurons)	GluA1	decrease	hippocampus		Mao et al., 2015
ASD	Shank1 (KI R882H)	GluA2	increase	hippocampus	decrease social recognition memory	Qin et al., 2022
ASD	Shank2	GluA1	decrease	hippocampus	decrease sociability and social recognition memory	Peter et al., 2016
ASD	Shank2 (forebrain overexpression)	GluA2	decrease	hippocampus	decrease sociability and nesting behavior	Eltokhi et al., 2021
ASD	Shank3 (ACC KD)		decrease mEPSC	ACC	decrease sociability and social recognition memory	Guo B. et al., 2019
ASD	Shank3 haploinsufficiency	GluA1	decrease	hippocampus	decrease social interaction, sociability	Bozdagi et al., 2010, Wang et al., 2011
ASD	Shank3 haploinsufficiency	GluA2	decrease	striatum	decrease social interaction and social recognition memory	Peca et al., 2011
ASD	Shank3 haploinsufficiency		decrease mEPSC	PFC and striatum	decrease sociability	Rapanelli et al., 2022
ASD	VPA		increase mEPSC	lateral amygdala	decrease social interaction	Lin et al., 2013
ASD	VPA		increase mEPSC	amygdala	decrease sociability	Wu et al., 2018
ASD	VPA	GluA2,3,4	increase	PFC and hippocampus	decrease social recognition memory	Mishra et al., 2022
ASD	VPA	GluA2,3,4	increase	PFC and hippocampus	decrease social recognition memory	Abhishek et al., 2022
ASD	VPA	GluA1,2	increase	PFC and hippocampus	decrease social interaction	Schiavi et al., 2022
ASD	VPA	GluA1	increase	PFC	decrease sociability and social recognition memory	Kim J. W. et al., 2019
ASD	VPA (early postnatal treatment)	GluA4	decrease	BLA		Wang et al., 2021
ASD	VRK3 KO		decrease Ca2+ permeable AMPARs		decrease sociability and social recognition memory	Kang et al., 2017
Depression	caspase-1 overexpression	GluA1	decrease	hippocampus	susceptible to social impairment after stress	Li et al., 2018
Depression	chronic restrain stress	GluA1,2	decrease	PFC	decrease sociability and social recognition memory, increase social dominance	Park et al., 2018
Depression	chronic social defeat stress	GluA1	decrease	hippocampus	decrease social interaction	Sakai et al., 2021
Depression	chronic social defeat stress	GluA2	decrease	hippocampus	decrease social interaction	Iniguez et al., 2016
Depression	chronic social defeat stress	GluA2	decrease	NAc	decrease social interaction	Vialou et al., 2010
Depression	chronic social defeat stress	GluA1	increase	NAc	decrease social interaction	Vialou et al., 2010
Depression	EphB2 KO	GluA1,2	decrease	PFC	susceptible to social impairment after stress	Zhang et al., 2016
Depression	GluA2 KI (K882A)	GluA2	decrease	hippocampus and NAc	susceptible to social impairment after stress	Ellis et al., 2017
Depression	neonatal isolation	GluA1	decrease	PFC	increase social dominance	Tada et al., 2016
Diabetes	streptozotocin-induced diabetes	GluA1	increase	hippocampus	decrease social recognition memory	Ueda et al., 2021a
Schizophrenia	miR-124-3p overexpression	GluA2	decrease, but higher mEPSC amplitude	PFC	decrease sociability and social recognition memory	Namkung et al., 2023
Schizophrenia	NVHL	GluA1	decrease	hippocampus and PFC	decrease social interaction and social recognition memory	Yabuki et al., 2019
Schizophrenia	S-SCAM (duplication)	GluA3	increase	hippocampus	decrease in sociability and social recognition memory	Zhang et al., 2015
Schizophrenia	Syndapin I KO	GluA1,2	decrease mEPSC	hippocampus and PFC	decrease social recognition memory	Koch et al., 2020

ACC, anterior cingulate cortex; AHI1, abelson helper integration site-1; ASD, autism spectrum disorder; BLA, basolateral amygdala; CNTNAP2, contactin-associated protein-like 2; EPAC2, Gpc4, glypican 4; IQSEC2, IQ motif and Sec7 domain 2; Lrfn2, leucine rich repeat and fibronectin type III domain containing 2; KI, knockin; KO, knockout; MDGL2, MAM domain containing glycosylphosphatidylinositol anchor 2; miR, microRNA; NOMA-GAP, neurite outgrowth multiadaptor RhoGAP; NAc, nucleus accumbens; NVHL, neonatal ventral hippocampal lesion; PFC, prefrontal cortex; PV, parvalbumin; S-SCAM, synaptic scaffolding molecule; Tph2, tryptophan hydroxylase-2; VPA, valproic acid induced model of ASD; VRK3, vaccinia-related kinase 3.

### 5.1 Role of GluA1 vs. GluA3

Examining the role of the AMPAR subunits revealed opposite effects of GluA1 and GluA3 in aggression. While knocking down GluA1 reduces aggression, GluA3 KO mice show enhanced aggressive behavior. The opposite roles played by these subunits on aggression are surprising since both subunits are commonly expressed in glutamate synapses. However, there are also differences in functional properties between GluA1- and GluA3-containing receptors. Compared to AMPARs lacking GluA3, AMPARs lacking GluA1 have lower conductance (Renner et al., 2017). Homomeric GluA1 AMPARs also have higher conductance than heteromeric AMPARs (Cull-Candy et al., 2006). Finally, GluA1 expression levels in synapses and extrasynaptic regions are much higher than GluA3 (Lu et al., 2009). Knocking down these subunits will have differing impacts on fast synaptic transmission. Furthermore, GluA1 and GluA3 have distinct roles in synaptic plasticity. For instance, the KO of GluA1 resulted in significant LTP impairment, which cannot be produced by the KO of GluA3 alone or together with the GluA2 subunit (Meng et al., 2003). In addition, pharmacologically raising cAMP levels with IBMS potentiated hippocampal extrasynaptic AMPAR currents in GluA1 KO, but not GluA3 KO, mice (Renner et al., 2017). Proteomic studies also revealed distinct interactomes between GluA1/2 and GluA2/3 AMPARs (van der Spek et al., 2022). Finally, the opposing role of GluA1 and GluA3 on social behaviors may be related to their differences in region-specific expression. While GluA1 is more abundant than GluA3 in the hippocampus, GluA3 levels in the cortex are higher than GluA1 (Schwenk et al., 2014; Pandya et al., 2017). In addition, GluA3 exhibits a developmental increase in expression around 2 weeks postnatal age, while GluA1 levels are similar across the first postnatal month. Further studies could examine the contribution of the synaptic currents and plasticity mediated by these subunits to aggression, especially GluA3 which is not as well understood as GluA1.

### 5.2 Role of AMPAR plasticity

Various findings suggest that the plastic properties of AMPARs are crucial for social behaviors. For instance, targeting GRIP1/2 and the phosphorylation sites of GluA2 S880 (Han et al., 2017), both implicated in AMPAR trafficking, could impair social memory (Bariselli et al., 2018). The detection of a novel conspecific is associated with the insertion of GluA2-lacking AMPARs into glutamate synapses on the VTA (Bariselli et al., 2018). While inhibiting GluA2-lacking AMPAR abolished this novelty-driven plasticity and impaired social novelty preference, upregulation of GluA2-lacking AMPAR also leads to social deficits. How these plastic processes regulate social information processing is less clear. As the insertion of GluA2-lacking AMPARs was observed after LTP induction (Wenthold et al., 1996; Brorson et al., 2004; Ziff, 2007; Kamalova and Nakagawa, 2021), LTP-related processes may mediate the detection of a novel social target (Li et al., 2003). The role of hippocampal LTP and LTD in the detection of the novelty of contextual information has been investigated. While the exposure to an empty novel context facilitated LTP, LTD is enabled by the presence of novel objects (Kemp and Manahan-Vaughan, 2007). Whether LTP and LTD are similarly regulated by the presence of novel social targets remains unclear. Social novelty detection is also related to the retrieval of social memory for familiar social targets, which is mediated by the enhanced activity of engram cells in both the dorsal and ventral hippocampus (Okuyama et al., 2016; Zhang et al., 2019). Since LTP and LTD could modulate the activity of engram cells (Frankland et al., 2019), these plastic changes may modulate mouse performance in the social novelty test by regulating social memory retrieval. Notably, NMDARs and metabotropic glutamate receptors, which are upstream players that regulate the plastic properties of AMPARs, have been shown to regulate social behaviors (Jacobs and Tsien, 2017; Zoicas and Kornhuber, 2019; Yang et al., 2020; Chen et al., 2022; Zhang et al., 2022). With our wealth of knowledge on the induction and expression mechanisms of synaptic plasticity and the animal models and experimental approaches that allow us to distinguish the behavioral outcomes of synaptic plasticity (Kessels and Malinow, 2009; Magee and Grienberger, 2020), examining the roles of AMPAR plasticity in the processing and storage of social information is a promising research avenue.

Although the role of homeostatic plasticity in social behavior is not clear, findings from animal models of brain disorders suggest its involvement in the regulation of social behaviors. Social deficits have been reported in Fragile X syndrome, an ASD that is related to the mutation of FMR1 gene. Animal models of fragile X syndrome showed reduced expression of fragile X mental retardation protein and GluA1 expression, which leads to defective scaling-up (Soden and Chen, 2010). In addition, defective homeostatic plasticity is associated with memory impairment in Alzheimer’s disease (Styr and Slutsky, 2018), which also displayed deficits in social behaviors. These findings warrant more studies of the role of homosynaptic plasticity in the regulation of social behaviors.

### 5.3 Future directions

Although rodent models have been extensively used for studying social behaviors, most of these studies focus on sociability and social novelty detection. While these studies are crucial for our understanding of social recognition memory, other aspects of social behaviors that could be examined in rodents are largely ignored. For instance, emotional valence is commonly associated with social experience (Padilla-Coreano et al., 2022). Increasing findings have revealed the neural mechanisms of the processing and storage of social information of different valences, also known as social valence. How AMPARs regulate social valence remains unclear. Apart from the recognition of novel conspecifics, social behaviors also require the recognition of individual familiar conspecifics for establishing more complex social roles, such as kinship and social hierarchy. By associating individual social targets with either positive (Kong et al., 2023) or negative valence (Lai et al., 2005), rodents can distinguish two familiar conspecifics to reveal individual recognition. AMPARs could be crucial for determining the right social responses by identifying distinct social characteristics from individuals. Finally, since the social transmission of olfactory cues of food (Loureiro et al., 2019) or emotional status of conspecific (Shi et al., 2022) has been shown to be mediated by synaptic plasticity, further investigating the role of AMPARs in social behaviors could reveal synaptic mechanisms of higher social functions such as social communication (Chen et al., 2021; Ebbesen and Froemke, 2021) and empathy (Kim A. et al., 2019).

Like other fields of behavioral science, our understanding of the neural mechanisms of social behaviors is based largely on findings using male animals. Since recognizing conspecifics is important for social animals of both sexes, both male and female rodents share similar social behaviors. For instance, both male and female rodents prefer to interact with novel social targets than nonsocial objects (An et al., 2011; Cavigelli et al., 2011; Perkins et al., 2016). Male and female rodents also displayed a preference for interacting with a novel vs. a familiar conspecific (Moy et al., 2004; Markham and Juraska, 2007). However, social interaction can be enhanced with conspecifics of the opposite sex (Kopachev et al., 2022). In addition, several properties of social behaviors are different between male and female rodents. Female rodents interact with social targets slightly but significantly less frequently than male rodents (Markham and Juraska, 2007; Karlsson et al., 2015), especially after prolonged social interaction (Netser et al., 2017). Moreover, female mice performed poorer than male mice in a more complex version of the social novelty test to identify a familiar mouse from 4 other novel mice (Krueger-Burg et al., 2016). On the other hand, in Fragile X syndrome model, female mice exhibited no social impairment while male mice displayed deficits in sociability (Marsillo et al., 2021). Finally, as the trafficking and/or the function of AMPARs can be regulated by female (Foy et al., 2010; Sparsa et al., 2010) and male gonadal hormones (Ziehn et al., 2012), these receptors could be crucial for social behaviors between individuals from the same sex or opposite sexes. Further development of animal models for studying individual recognition and dyadic behaviors between individuals of different sexes are needed.

As AMPAR changes clearly contribute to some abnormal social behaviors in brain diseases, targeting AMPARs is a promising approach for restoring normal social function. For instance, PAM, such as ampakine, has been successfully used to elevate AMPAR function and social behaviors in animal models of ASD (Choi et al., 2022). With the advancement of PAM designs that are based on the structural properties of different AMPAR subunits (Frydenvang et al., 2022), PAMs could be a promising approach to address the deficiency of specific AMPAR subunits. PAMs that preferentially facilitate GluA3 subunit function, such as PEPA (Sekiguchi et al., 1997), could be tested for their impact in attenuating aggression that is associated with reduced GluA3 expression. Although most of the reviewed studies focused on GluA1-3, GluA4 may also have significant contribution to social behaviors and social deficits in brain disorders. Despite its expression subsiding in the forebrain after development, GluA4 expression remains high in interneurons, especially the PV subtype. Notably, abnormality in interneuron functioning has been highly implicated in brain disorders that displayed social deficits, including ASD and schizophrenia (Nakazawa et al., 2012; Contractor et al., 2021). In addition, GluA4 is highly expressed in the cerebellum, which is also known to contribute to social cognition. Further studies of the role of this AMPAR subunit in social behaviors is therefore needed. In addition, developing drugs targeting GluA4 could be another interesting research avenue to study its regulation of social behaviors, which can in turn be useful for treating deficits in these behaviors in brain disorders. Clinically approved negative allosteric modulators of AMPAR, such as perampanel (Pandey et al., 2010), could also be used for treating social deficits that are associated with enhanced AMPAR function. Apart from the channel-forming subunits, auxiliary subunits could be targeted for modulating AMPAR functions. TARPs have been shown to modulate the trafficking and gating properties of AMPARs (Milstein and Nicoll, 2008). Recent findings have shown that injecting TARP γ8 ligand JNJ-61432059 into mouse BLA or ventral hippocampus could reduce and enhance GluA1- and GluA2-containing AMPARs, respectively (Zhang et al., 2023), and reduce repetitive alcohol self-administration, in an animal model of addiction (Hoffman et al., 2021) (for review, see (Brogi et al., 2019)). Taken together, these AMPAR-modulating reagents could be promising therapeutics for restoring normal social functioning in brain diseases.

## 6 Conclusion

As the most abundant ionotropic glutamatergic receptors in the brain, it is not surprising that AMPAR expression, function and plasticity are closely related to different types of social behaviors. Indeed, examining the role of different AMPAR subunits has revealed opposing roles of GluA1 and GluA3 in the expression of aggressive behaviors. Plastic changes of GluA2-containing and -lacking AMPARs, which correspond to distinct functional properties such as calcium permeability and inward rectification, have been shown to be crucial elements for social cognition, such as novelty detection. Not surprisingly, aberrant AMPAR-mediated synaptic transmission has been associated with impaired social behaviors and cognition in brain diseases. These findings highlight the importance of further investigating the role of AMPAR in social behaviors. Promising research avenues include the role of distinct subunits like TARPs and GluA3 in social cognition. Given the distinct roles of AMPAR subunits in regulating social behaviors, developing modulators of specific AMPAR subunits or combinations is warranted not only for understanding their still largely unknown roles in social behaviors, but for using them in treating social deficits in brain disorders.

## Author contributions

QX: Writing−original draft, Writing−review and editing. AL: Writing−original draft, Writing−review and editing. TW: Conceptualization, Funding acquisition, Supervision, Writing−original draft, Writing−review and editing.
